# The relationship between amount of extra-prostatic extension and length of capsular contact: performances from MR images and radical prostatectomy specimens

**DOI:** 10.3906/sag-2012-55

**Published:** 2021-08-30

**Authors:** Aslıhan ONAY, Barış BAKIR

**Affiliations:** 1 Department of Radiology, Faculty of Medicine, TOBB ETÜ University, Ankara Turkey; 2 Department of Radiology, Medical Faculty, İstanbul University İstanbul School of Medicine, İstanbul Turkey

**Keywords:** Prostate cancer, extraprostatic extension, length of capsular contact, tumor grading, multi-parametric magnetic resonance imaging

## Abstract

**Backround/aim:**

In prostate cancer, extraprostatic extension (EPE) is an unfavorable prognostic factor, and the grade of EPE is correlated with the prognosis. This study aims to evaluate the utility of length of capsular contact (LCC ) in predicting the grade of EPE by correlating the measurements from MRI images and the measurements performed from radical prostatectomy specimens.

**Materials and methods:**

MR images and specimens of 110 tumors are analyzed retrospectively. The specimens are used as reference to validate the presence of EPE and to measure the ground truth LCC. MR images are evaluated by two radiologists to identify the presence of EPE and to predict the LCC indirectly. Reliability, accuracy, sensitivity, and specificity of the evaluations are analyzed in comparison with the findings obtained from the specimens.

**Results:**

In detection of EPE existence, the radiologists achieve almost the same performance (all AUCs = 0.73) with optimal cut-off values lead to moderate sensitivity and specificity pairs (For cut-off = 15.8 mm; Se = 0.69, Sp = 0.68 and for cut-off of 14.5 mm: Se = 0.77, Sp = 0.62). In distinguishing high-grade EPE from low-grade EPE, the radiologists accomplish very similar performances (AUCs = 0.73 and 0.72) Optimal thresholds of 20.0 mm and 18.5 mm for the readers retrospectively reveal medium sensitivity and specificity pairs (Se = 0.64, Sp = 0.67; Se = 0.64, Sp = 0.67).

**Conclusion:**

Consistent LCC estimates can be obtained from MR images providing a beneficial metric for detecting the existence of EPE and for discriminating the grades of EPE.

## 1. Introduction 

Accurate local staging of prostate cancer is crucial for determining the prognosis and establishing the best treatment plan [1–3]. The staging is highly influenced by the status of extraprostatic extension (EPE). Moreover, a greater EPE is associated with a significant prognosis of the disease. It has also an impact on surgical strategy by modifying the surgical technique, i.e. performing a wider margin of excision versus narrower margin of excision that depends on the amount of EPE. In the case of a high amount of EPE, the patient can be informed about the increased risk of positive surgical margin, and neoadjuvant therapy can be considered. Besides, such a patient can be treated with radiation therapy or hormone therapy before surgery or instead of surgery. In detecting EPE and determining the amount of EPE from pathology specimens, several sub-classification methods are proposed [4–7]. However, there has been no common consensus for the optimal method and sub-classification categories are exempted from the 2010 tumor node metastasis (TNM) staging system [8]. In addition to this, there is a great need for a less complicated and easy to use technique to detect EPE presence and to predict the amount of EPE.

Multi-parametric magnetic resonance (MR) imaging is the most favorable imaging technique for local staging of prostate cancer [9–11] and also offers many imaging findings linked to EPE. When compared to the findings from clinical examination, the findings from the images are demonstrated to be more beneficial in expressing EPE [12,13]. Nevertheless, a recent meta-analysis shows that MR imaging-based local staging of prostate cancer shows high specificity with low sensitivity [14]. ‘A tumor-capsule interface of greater than 1.0 cm’ is an MR imaging finding introduced in the prostate imaging-reporting and data system version 2.1 (PIRADS V2.1) guideline that linked to EPE [15]. The tumor-capsule interface, also named as the length of capsular contact, measured as the tumor contact length with prostate capsule on the images establishes a good agreement and performance [16–22]. However, the relationship between the length of capsular contact and the amount of EPE has not been understood fully yet. 

The current study aims to figure out the utility of length of capsular contact (LCC) from MR images in detecting and grading the extraprostatic extension (EPE) for prostate tumors in comparison with the measurements performed on radical prostatectomy specimens.

## 2. Materials and methods

### 2.1. MR Imaging of the prostate and radical prostatectomy intervention

Institutional review board approval and informed consent are secured for this retrospective study. A search on the electronic databases at our institution explored a total of 121 prostate tumors from 121 patients who underwent MR imaging before radical prostatectomy intervention. Unreachable pathology records were of concern for four tumors. The time interval between the imaging and the RP was longer than six months for five cases. MR images of the two tumors were with severe artefacts. These tumors were excluded from the study and the remaining 110 tumors were taken into consideration (various imaging features from these tumors were reported in our previous work focused on assessment of the grade of extraprostatic extension of the prostate carcinoma [23]; however, the current study targets the length of capsular contact feature for the first time). In addition, a portion of the study population (approximately 70%) was used to evaluate whether the International Society of Urological Pathology (ISUP) grade group of the tumors influenced the relationship between LCC and EPE presence (ref); however, for the first time, we analyzed the role of LCC in assessment of the amount of EPE with a larger study group in our current study.

MR imaging of the prostate is conducted with a 3T MRI scanner (Magnetom Skyra, Siemens Medical Solutions, Erlangen, Germany) and a sixteen-channel phased-array surface coil in a multi-parametric manner. To reduce motion artifacts associated with bowel peristalsis, imaging is performed after intramuscular injection of 20 mg of butylscopolamine (Buscopan, Boehringer). The imaging protocol respectively consists of T2-weighted imaging (T2WI), fat-suppressed dynamic contrast-enhanced imaging (DCE), free-breathing diffusion-weighted imaging (DWI), and apparent diffusion coefficient (ADC) mapping with the imaging parameters listed in Table 1. 

**Table 1:** MR imaging sequences and sequence dedicated parameter values are summarized.

Sequence	Imaging plane	TR/TE (ms)	FOV (mm2)	ST/Gap (mm)	Matrix size
T2W (TSE)	Axial, Coronal and Sagittal	3566–3631/100	200 × 200	3.0	512 × 352
DCE (GRE)	Axial	4.86/1.76	260 × 260	3.6	192 × 154
DWI (SS-EPI)	Axial	4000/101	260 × 260	3.6/0.3	192 × 154

TR: Repetition time, TE: Echo time, FOV: Field of view, ST: Slice thickness, TSE: Turbo spin echo, GRE: Gradient recalled echo, SS-EPI: Single-shot echo-planar imaging with b-values of 0, 50, 100, 200, 400 and 800 s/mm2 with automatic apparent diffusion coefficient mapping and computed high b-value mapping for b = 1500 s/mm2.

Following imaging, radical prostatectomy interventions are performed. All of the specimens gathered are fixed with 10% buffered neutral formalin, then surgical margins are painted with ink. The entire prostate gland and seminal vesicles are step sectioned from apex to base at 3–4 mm intervals in the plane perpendicular to the long axis of the prostate gland, and hematoxylin and eosin are used to stein these sections. An index lesion is marked according to the following benchmarks; 1: the prostate tumor foci that show EPE, 2: the prostate tumor foci that have the highest ISUP grading score, 3: the tumor foci that have the largest dimension. 

### 2.2. Prediction of the length of capsular contact 

For an index lesion identified, the absence or presence EPE and the pathologic length of tumor capsule contact on the RP specimen (p-LCC) are determined by an experienced uropathologist. In the presence of EPE, the pathological radial distance of EPE (p-RD), defined as the length of tumor protrusion perpendicular to the outer margin of the prostatic stroma, is measured additionally (in the existence of multiple foci of EPE, the measurement is done from the focus with the maximum extension). The index lesion is matched with a histopathological diagram as reference standard with the consensus of the uropathologist and two radiologists by taking into account of alterations on the shape and size of the prostate caused by the preservation of specimens. 

The two radiologists have 12 and 5 years of experience in genitourinary radiology (B.B and A.O). They independently explore all MR images using DynaCAD prostate software (version 3.3, Philips Healthcare). Each radiologist identifies the dominant prostate tumor foci with low signal intensity on ADC maps and high signal intensity on high b-value DWI images with or without early contrast enhancement on DCE images. Next, the radiologist measures the length of the tumor capsule interface (MR-LCC) on the axial T2W image according to the method described by Baco et al [18] using the curved measurement tool offered by the software. If the radiologist cannot manage to identify any contact between the tumor and the capsule on the images, MR-LCC is considered to be zero. During measurements, the radiologists are aware that the patients have prostate cancer verified by radical prostatectomy, but they are blinded to the demographical, clinical, and final pathology findings.

### 2.3. Statistical analysis

Mann–Whitney U test or independent-samples t-test is conducted to detect significant differences in the LCC estimates for the tumors with and without EPE and for the tumors having high and low grades of EPE. Spearman Rho (ρ) is used to assess the correlation between the p-RD and the LCC estimates and between p-LCC and the LCC estimated from MR images. The inter-observer agreement for the MR-LCC estimates across the radiologists is determined using the intraclass correlation coefficient (ICC). Performances of the LCC estimates in the diagnosis of EPE and in distinguishing the low-grade EPE from high-grade EPE are obtained by performing receiver operator characteristic curve analyses and by calculating the area under the curves (AUC). Youden analysis is implemented to obtain the optimal threshold for the LCC, and the sensitivity (Se) and specificity (Sp) are reported for that threshold. A *p*-value of <0.05 is considered for statistical significance. All analyses are performed using IBM SPSS for Windows (v25; IBM Corp., Armonk, NY, USA).

## 3. Results 

Radical prostatectomy specimens and multi-parametric MR images of 110 prostate tumors diagnosed with prostate cancer are evaluated retrospectively. The mean time interval between the imaging and the intervention is 73.1 days (range: 11–192 days). Organ-confined disease is acknowledged for 84 tumors, and EPE is detected for the remaining 26 tumors from the specimens. The radial distance of the extension (p-RD) measured from the specimens of the EPE positive tumors gives a median value of 1.0 mm that is later used as a cut-off to categorize the high-grade and low-grade EPE positive tumors. Consequently, among the 26 EPE positive tumors, 15 tumors are figured out to be with low-grade EPE and 11 tumors are with high-grade EPE. Figures 1a–1c and Figures 2a–2c demonstrate the measurements for the two representative tumors from the study dataset. Tumor localizations in the RP specimens of the same cases are shown in Figure 1d and Figure 2d.

**Figure 1a:**
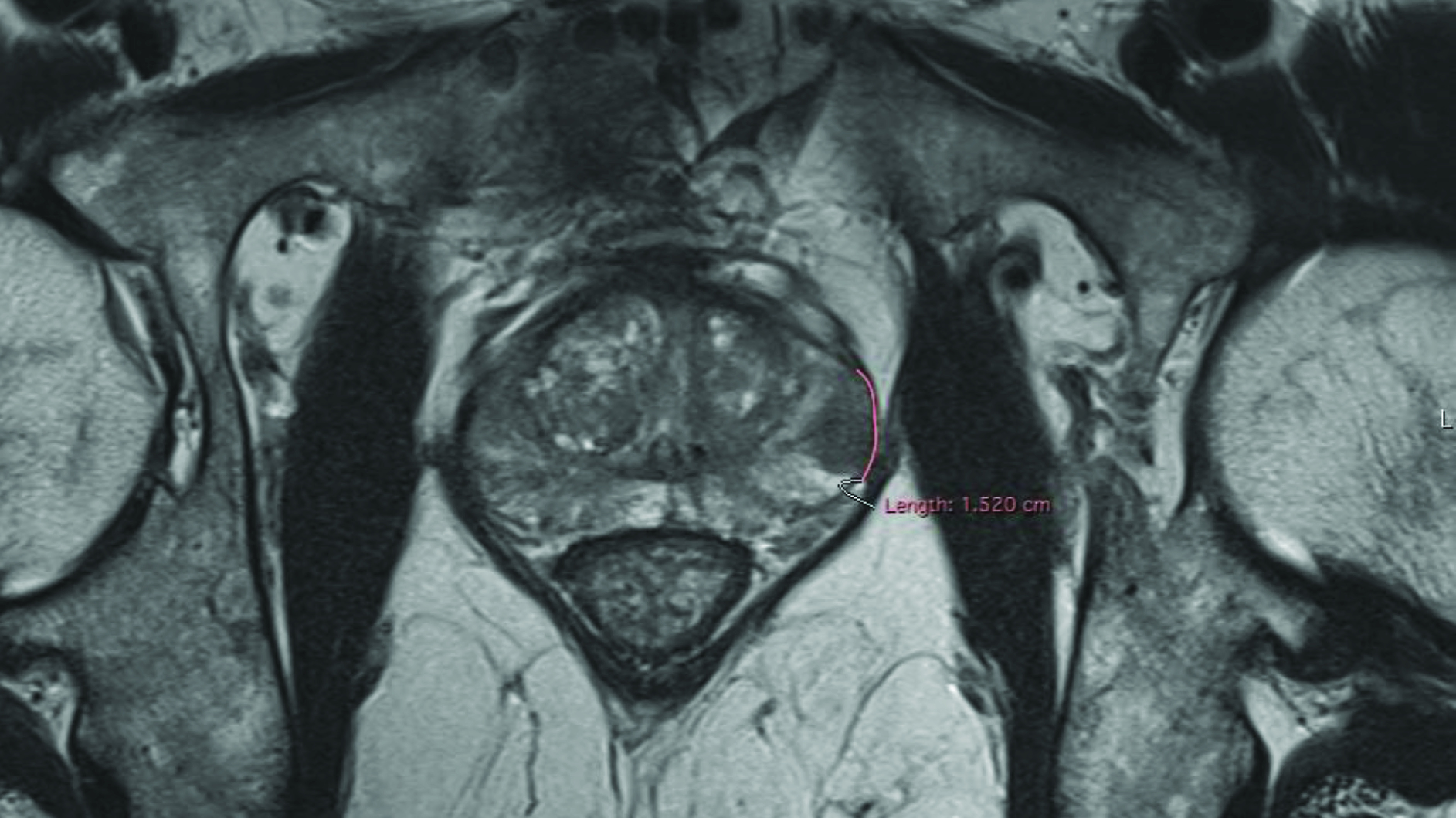
A prostate tumor on the left lateral peripheral zone with a Gleason score of 3+4 is seen. Axial T2 weigheted image shows the index lesion matched with radical prostatectomy specimen. Pathological analyses revealed p-RD = 0.5 mm and p-LCC = 15 mm while the radiologists respectively report MR-LCC1 = 15.2 mm and MR-LCC2 =16.0 mm.

**Figure 1b:**
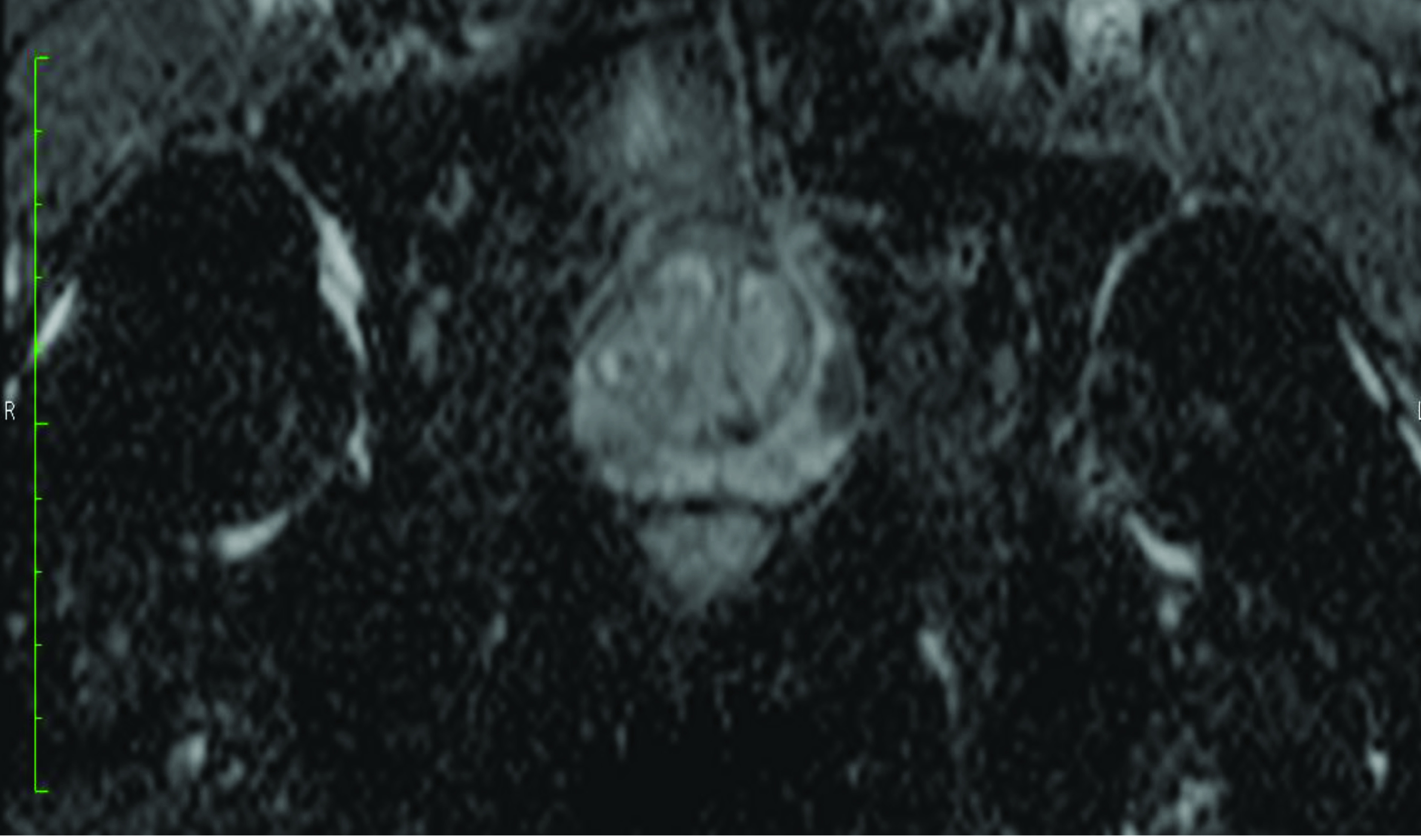
A prostate tumor on the left lateral peripheral zone with a Gleason score of 3+4 is seen. Axial ADC map shows the index lesion matched with radical prostatectomy specimen Pathological analyses revealed p-RD = 0.5 mm and p-LCC= 15 mm while the radiologists respectively report MR-LCC1 = 15.2 mm and MR-LCC2 =16.0 mm.

**Figure 1c:**
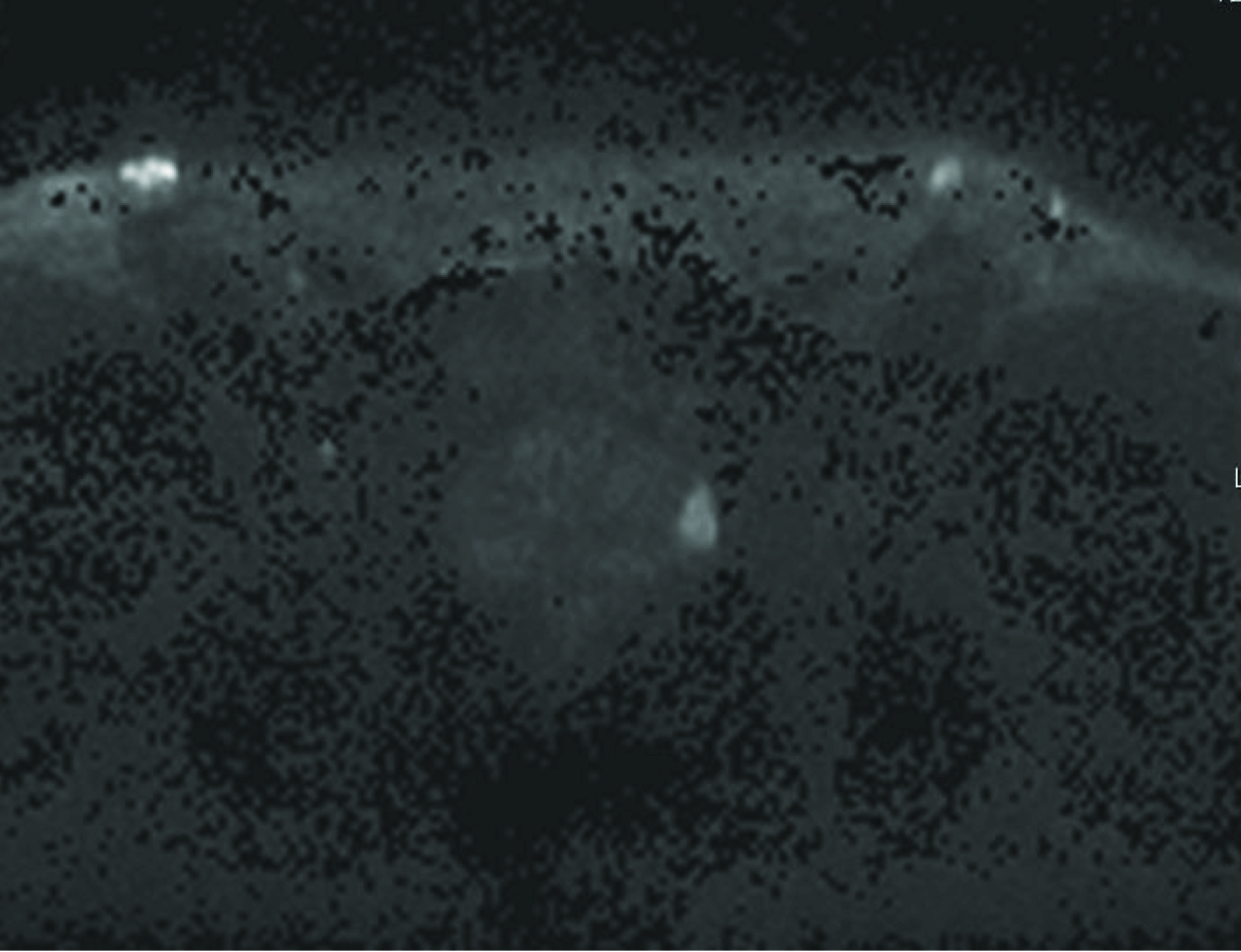
A prostate tumor on the left lateral peripheral zone with a Gleason score of 3+4 is seen. Axial high b-value computed diffusion-weighted image shows the index lesion matched with radical prostatectomy specimen. Pathological analyses revealed p-RD = 0.5 mm and p-LCC = 15 mm while the radiologists respectively report MR-LCC1 = 15.2 mm and MR-LCC2 =16.0 mm.

**Figure 1d:**
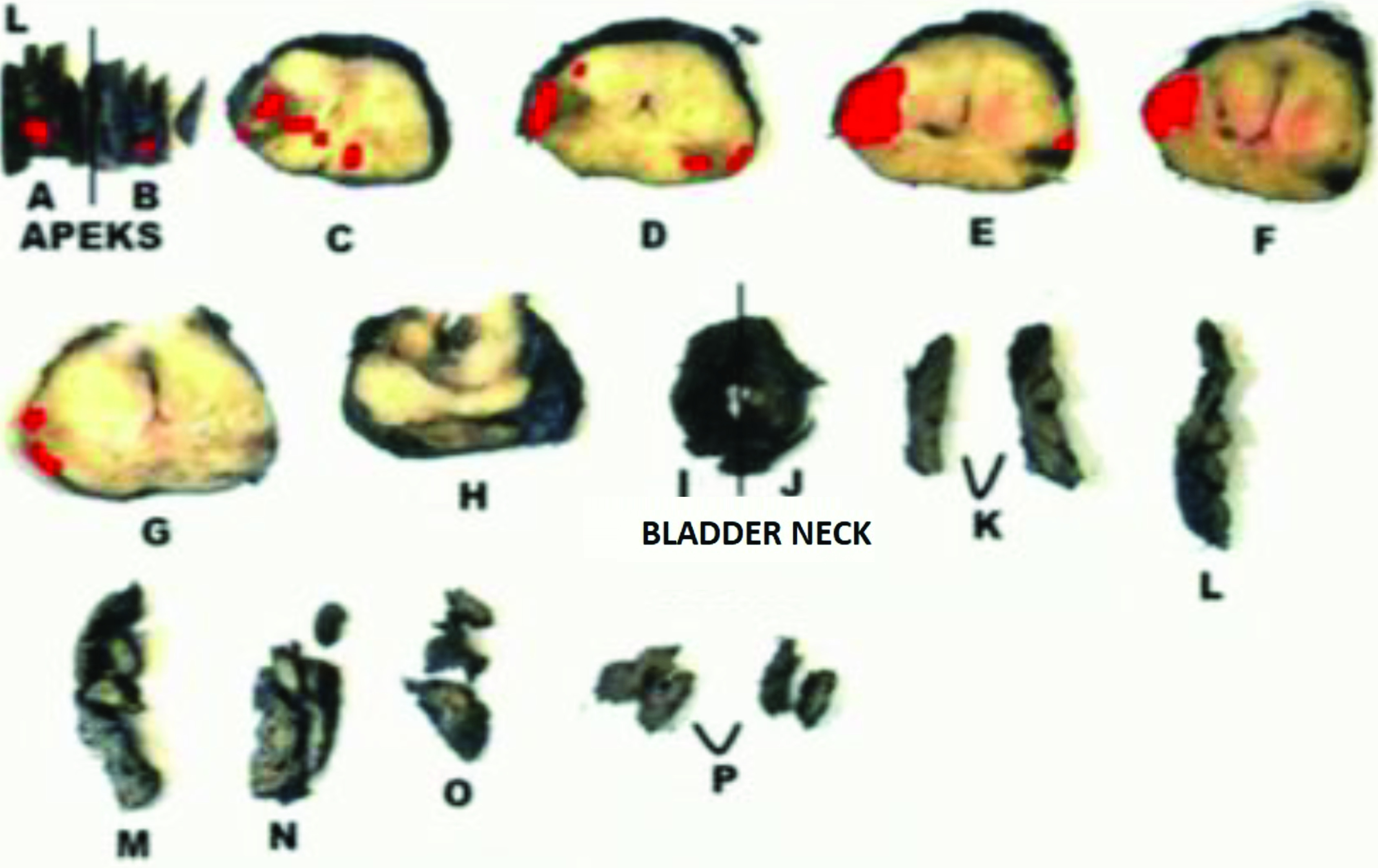
A prostate tumor on the left lateral peripheral zone with a Gleason score of 3+4 is seen. Schematic view of radical prostatectomy specimen demonstrates the index lesion Pathological analyses revealed p-RD = 0.5 mm and p-LCC = 15 mm while the radiologists respectively report MR-LCC1 = 15.2 mm and MR-LCC2 =16.0 mm.

**Figure 2a:**
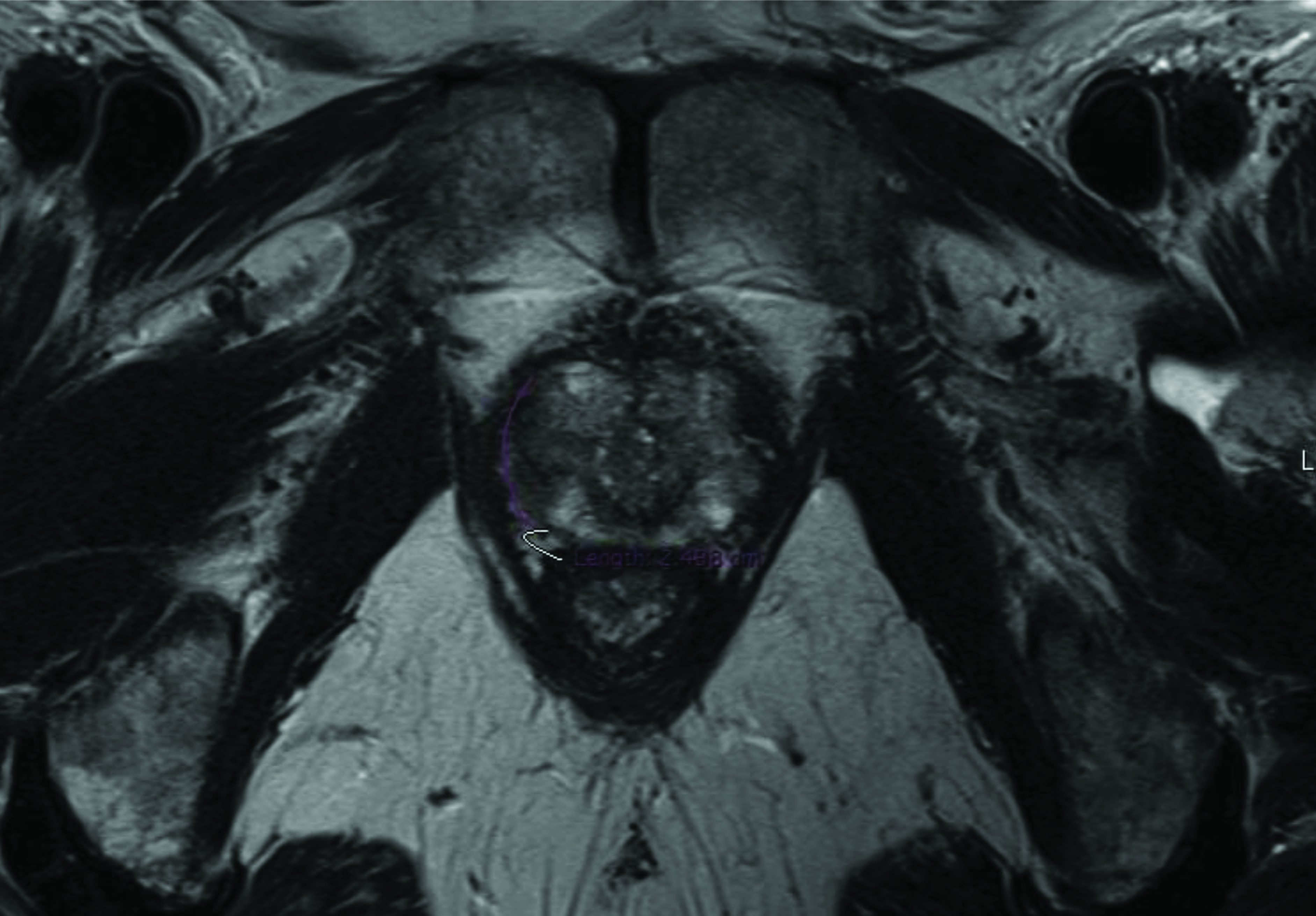
A prostate tumor on the right lateral peripheral zone with a Gleason score of 4+3 is given. Axial T2 weigheted image shows the dominant tumor foci verified with pathology. Pathological analyses revealed p-RD = 1.7 mm and p-LCC = 25.0 mm while the radiologists respectively report MR-LCC1 = 24.8 mm and MR-LCC2 = 24.0 mm.

**Figure 2b:**
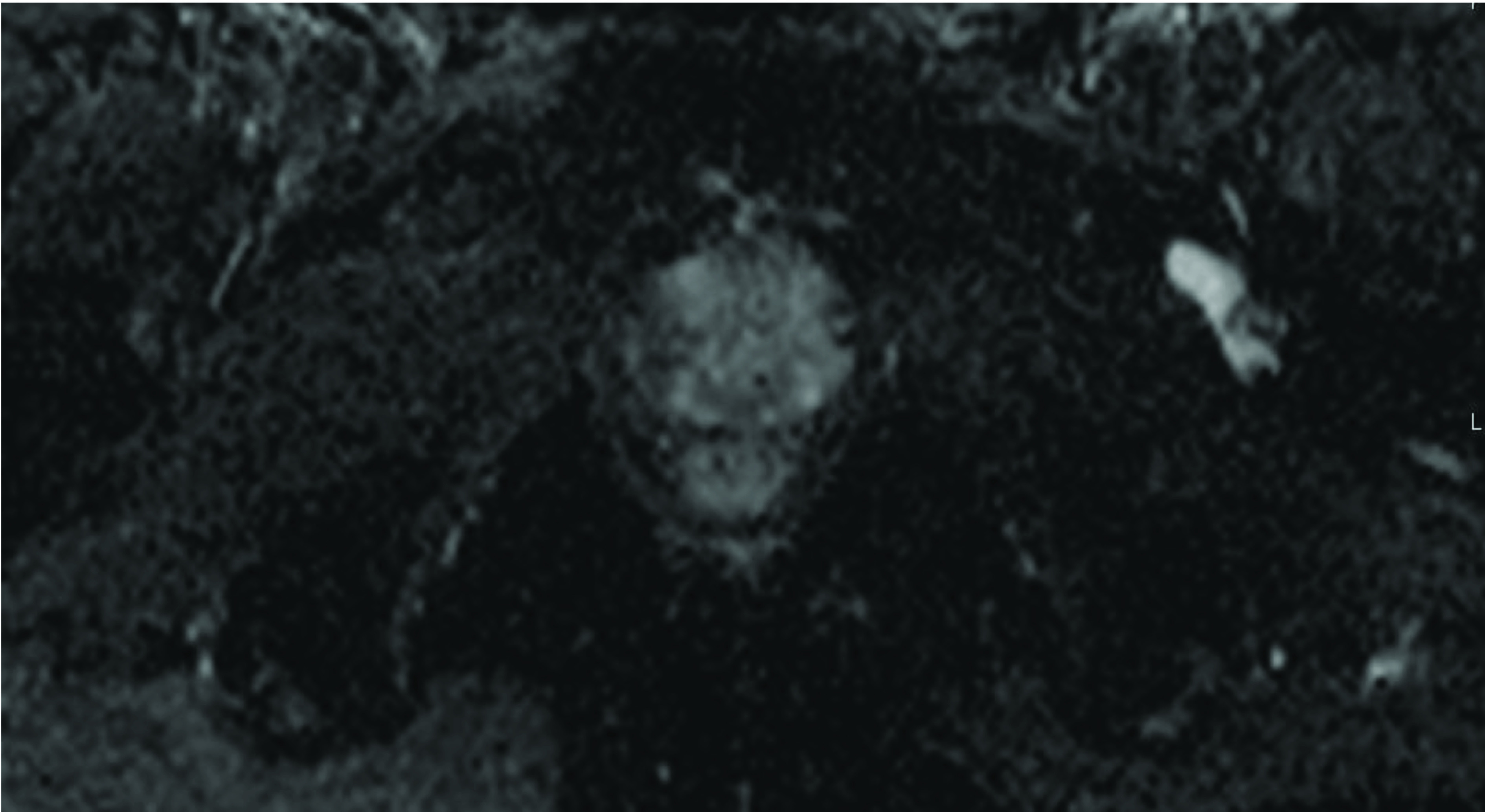
A prostate tumor on the right lateral peripheral zone with a Gleason score of 4+3 is given. Axial ADC map shows the dominant tumor foci verified with pathology. Pathological analyses revealed p-RD = 1.7 mm and p-LCC = 25.0 mm while the radiologists respectively report MR-LCC1 = 24.8 mm and MR-LCC2 = 24.0 mm.

**Figure 2c:**
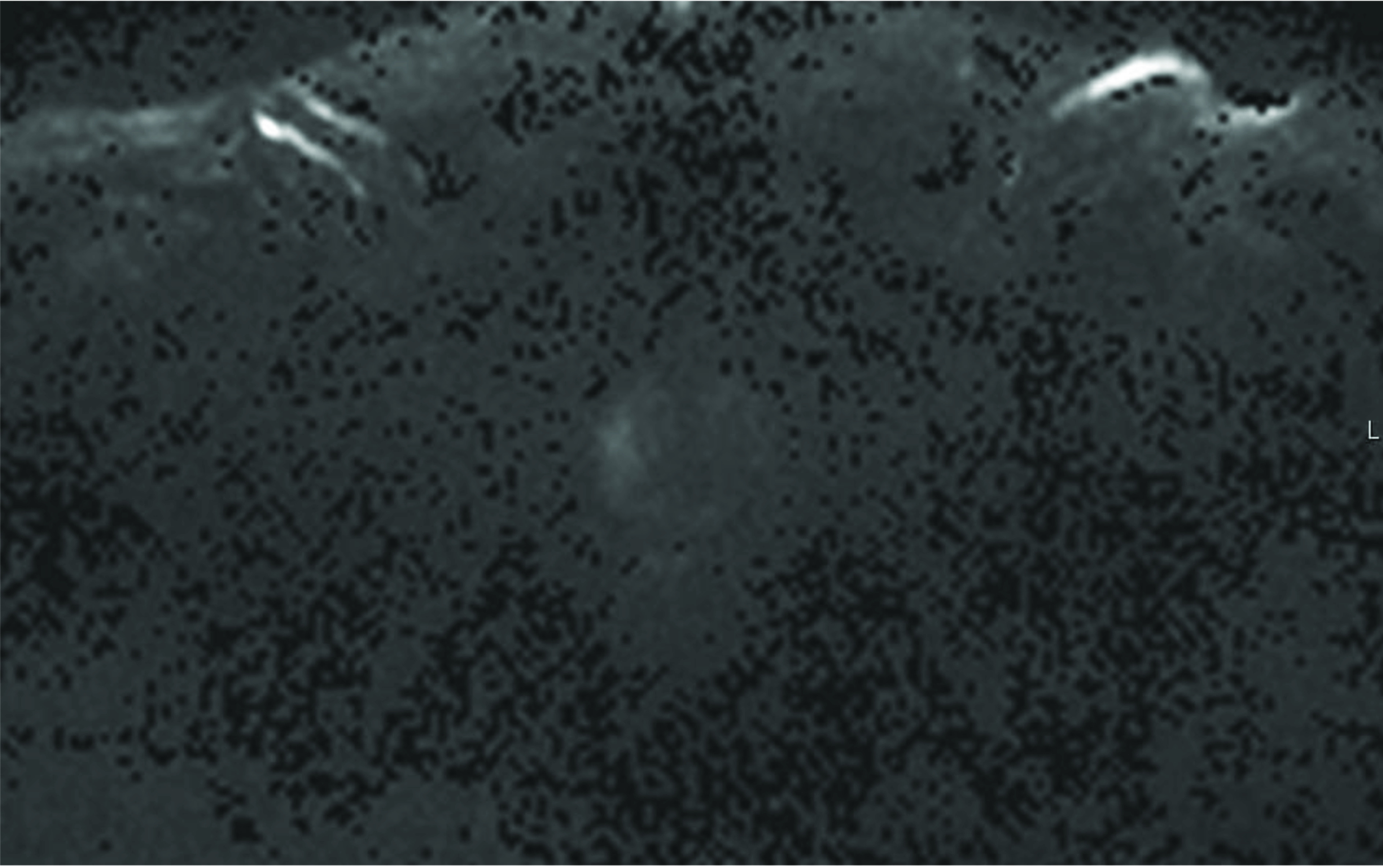
A prostate tumor on the right lateral peripheral zone with a Gleason score of 4+3 is given. Axial high b-value computed diffusion-weighted image shows the dominant tumor foci verified with pathology. Pathological analyses revealed p-RD = 1.7 mm and p-LCC = 25.0 mm while the radiologists respectively report MR-LCC1 = 24.8 mm and MR-LCC2 = 24.0 mm.

**Figure 2d:**
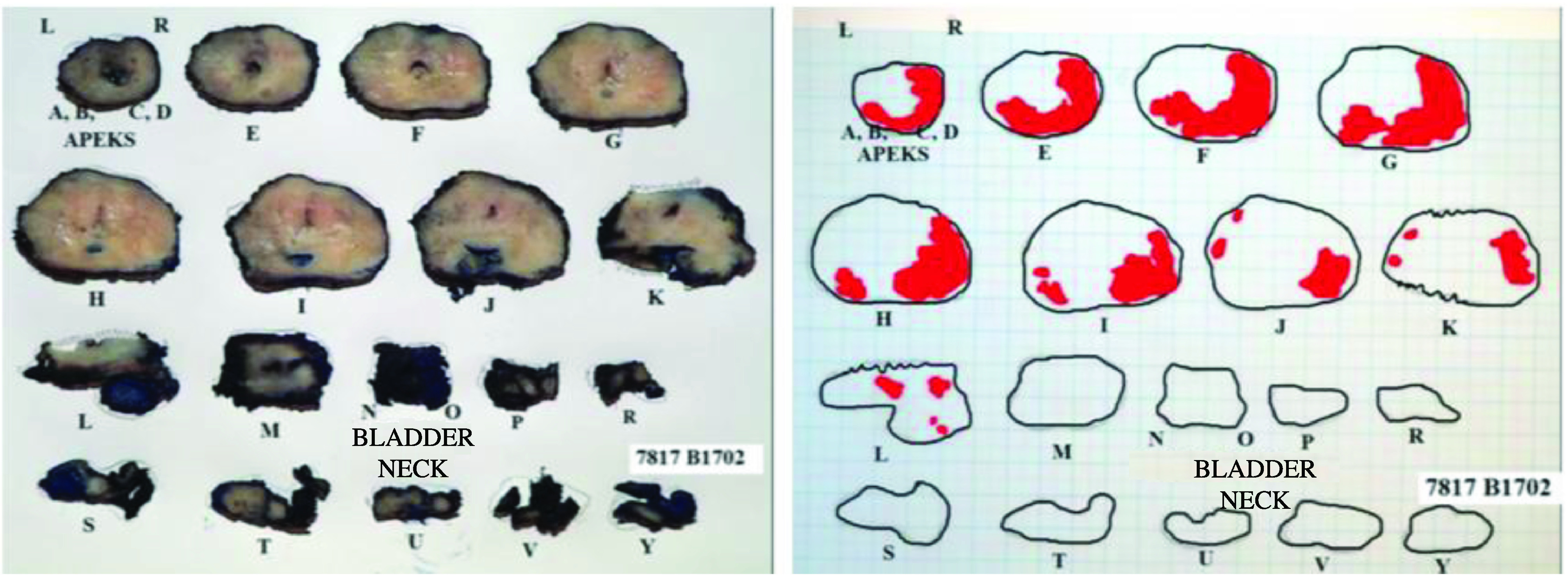
A prostate tumor on the right lateral peripheral zone with a Gleason score of 4+3 is given. Schematic view of radical prostatectomy specimen shows the dominant tumor foci verified with pathology. Pathological analyses revealed p-RD = 1.7 mm and p-LCC = 25.0 mm while the radiologists respectively report MR-LCC1 = 24.8 mm and MR-LCC2 = 24.0 mm.

**Figure 3:**
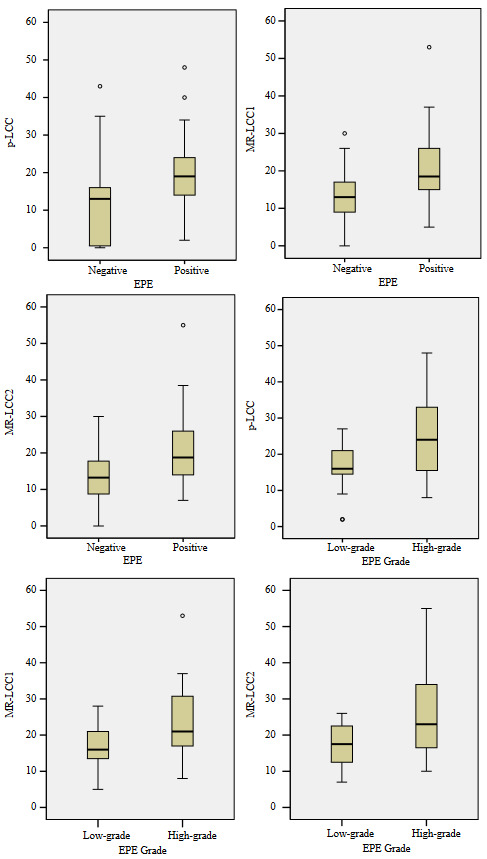
Boxplot for p-LCC from EPE negative and EPE positive tumors are demonstrated. Significant differences are present for p-LCC (p < 0.0001) between EPE negative and EPE positive tumors. b. Boxplot for MR-LCC1 from EPE negative and EPE positive tumors are demonstrated. Significant differences are present for MR-LCC1( p < 0.0001) between EPE negative and EPE positive tumors. c. Boxplot for MR-LCC2 from EPE negative and EPE positive tumors are demonstrated. Significant differences are present for MR-LCC2 (p < 0.0001) between EPE negative and EPE positive tumors. d. Boxplot for p-LCC from low-grade and high-grade EPE positive tumors are shown. Significant differences are present for p-LCC (p = 0.039) between low-grade and high-grade EPE positive tumors. e. Boxplot for MR-LCC1 from low-grade and high-grade EPE positive tumors are shown. Significant differences are present for MR-LCC1 (p = 0.032) between low-grade and high-grade EPE positive tumors. f. Boxplot for MR-LCC2 from low-grade and high-grade EPE positive tumors are shown. Significant differences are present for MR-LCC2 (p = 0.044) between low-grade and high-grade EPE positive tumors.

Average p-LCC and MR-LCC estimates from all the tumors taken into analyses are listed in Table 2, and conforming boxplots are presented in Figures 3a–3f). Both p-LCC, MR-LCC_1_ and MR-LCC_2 _is lower for the EPE negative tumors than the EPE positive ones (Figures 3a–3c) and an increase in both p-LCC, MR-LCC_1_ and MR-LCC_2_ is a precursor for high-grade EPE positive tumors (Figures 3d–3f). Significant differences are present for both p-LCC and MR-LCC between EPE negative and EPE positive tumors and also between low-grade and high-grade EPE positive tumors (p < 0.05 at all). Table 3 shows the correlations between MR-LCC and p-LCC from the tumors. For EPE negative tumors, moderate correlations are observable between p-LCC and MR-LCC estimates by the radiologists (ρ = 0.70 and 0.67, respectively) while slightly better correlations are noticeable between p-LCC and MR-LCCs for the EPE positive tumors (ρ = 0.72 and 0.67). Moderate correlations present between p-LCC and MR-LCCs both for low-grade EPE positive tumors (ρ = 0.67 and 0.62), but good correlations exist for high-grade EPE positive tumors (ρ = 0.82 and 0.74). Very strong correlations are noted between MR-LCC estimates by the radiologists for all EPE cases (ρ = 0.92–0.98). Overall, very similar MR-LCC estimates are obtained by the radiologists (ICC = 0.97, 95% CI = 0.96–0.98). On the other hand, p-RD shows a fair correlation with p-LCC (ρ = 0.39); however, moderate correlations are present between p-RD and MR-LCC estimates by the radiologists (ρ = 0.58 and 0.59, respectively) for the EPE positive tumors. All correlations are significant (p < 0.05). 

**Table 2:** The length of capsular contact measured from pathological specimens and estimated from MR images (in mm) are given.

	p-LCC	MR-LCC1	MR-LCC2
EPE negative	11.0 ± 9.1	12.5 ± 7.4	12.7 ± 7.5
EPE positive	20.0 ± 10.7	20.5 ± 10.3	21.0 ± 10.7
Low-grade	16.0 ± 7.2	16.7 ± 6.7	17.0 ± 6.5
High-grade	25.5 ± 12.5	25.3 ± 12.5	26.5 ± 13.1

**Table 3:** Correlations between the length of capsular contact estimated from MR images and measured from pathological specimens (Correlations are significant at p < 0.01) are shown.

	Spearman Rho (ρ) of MR-LCC1 vs p-LCC	Spearman Rho (ρ) of MR-LCC2 vs p-LCC	Spearman Rho (ρ) of MR-LCC1 vs MR-LCC2
EPE negative	0.70	0.67	0.97
EPE positive	0.72	0.67	0.96
Low-grade	0.67	0.62	0.92
High-grade	0.82	0.74	0.98

The performances of LCC in detecting the EPE positive tumors and in distinguishing the low-grade from high-grade EPE positive tumors are presented in Table 4, and the ROC plots obtained during analyses are as seen in Figure 4a and Figure 4b. In the detection of the EPE positive tumors, p-LCC performs fair (AUC = 0.74), and almost the same performance is accomplished by MR-LCC for both of the radiologists (all AUCs = 0.73) as shown in Figure 4a. For the optimal cut-off of 16.5mm, p-LCC reveals fair sensitivity and moderate specificity (Se/Sp = 0.58/0.77). For the optimal cut-off values of 14.5 mm and 15.8 mm for the radiologists, MR-LCC provides moderate sensitivity and specificity pairs (Se/Sp = 0.77/0.62 and Se/Sp = 0.69/0.68). Higher optimal cut-off is of concern for p-LCC achieving higher specificity but lower sensitivity when compared to the ones for MR-LCC. In distinguishing the low-grade from high-grade EPE positive tumors, almost the same fair performance is delivered by p-LCC and MR-LCC (AUCs= 0.71–73) as shown in Figure 4b. For the optical cut-off of 21.0 mm, p-LCC gives moderate sensitivity and specificity (Se/Sp= 0.64/0.73). The optimal cut-offs of 20.0 mm and 18.5 mm for MR-LCC reveal the same moderate sensitivity and moderate specificity (Se/Sp = 0.64/0.67 at all). Higher optimal cut-off is of concern for p-LCC achieving higher specificity when compared to the ones for MR-LCC.

**Table 4:** Performances of the LCC estimates are demonstrated.

		AUC (95% CI)	Cut-off (mm)	Se	Sp
In detecting EPE	p-LCC	0.74 (0.64–0.82)	16.5	0.58	0.77
	MR-LCC1	0.73 (0.62–0.84)	14.5	0.77	0.62
	MR-LCC2	0.73 (0.61–0.84)	15.8	0.69	0.68
In discriminating EPE grades	p-LCC	0.71 (0.49–0.94)	21.0	0.64	0.73
	MR-LCC1	0.73 (0.53–0.93)	20.0	0.64	0.67
	MR-LCC2	0.72 (0.52–0.92)	18.5	0.64	0.67

**Figure 4a:**
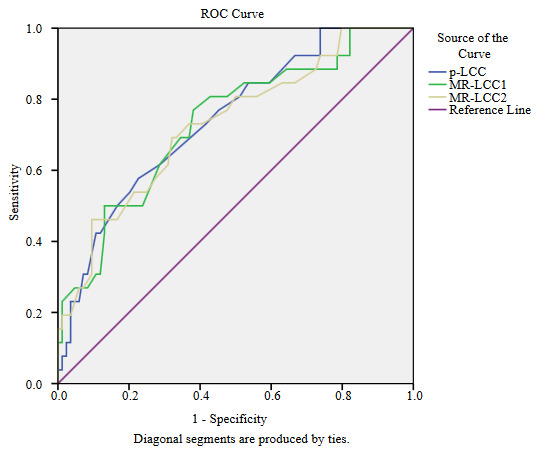
ROC plots for p-LCC, MR-LCC1, and MR-LCC2 in discrimination of EPE negative and EPE positive tumors are given. p-LCC (AUC = 0.74) and MR-LCC for both of the radiologists (all AUCs = 0.73) perform fair performance in the detection of the EPE positive tumors.

**Figure 4b:**
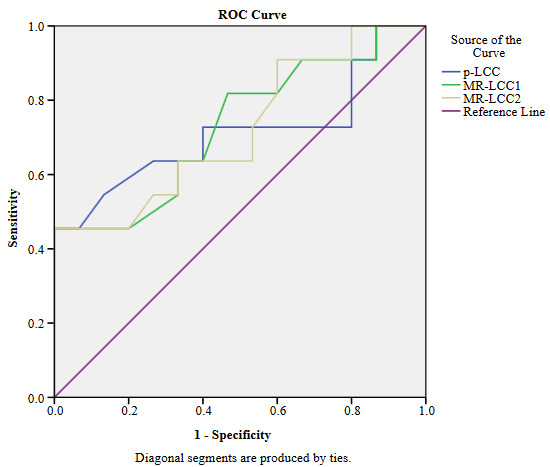
ROC plots for p-LCC, MR-LCC1, and MR-LCC2 in discrimination of low-grade and high-grade EPE positive tumors are given. Fair performance is also delivered by p-LCC and MR-LCC (AUCs = 0.71-73) in distinguishing the low-grade from high-grade EPE positive tumor

## 4. Discussion 

Detection and grading of extraprostatic extension (EPE) of prostate tumors are remarkably important for precise local staging of prostate cancer and management of the patients suffering from prostate cancer [4–7]. Multi-parametric MR images offer several metrics to improve the accuracy of prostate cancer staging. In the current study, the utility and the reproducibility of the length of capsular contact estimated from the multi-parametric MR images of the prostate tumors have been assessed for the purpose.

The length of capsular contact estimated from the multi-parametric MR images of the prostate tumors (MR-LCC) is reported to be the most prevalent and relatively objective imaging measure satisfying fair to good performances with good inter-reader agreements in detection of EPE existence [16,18–22]. Every 1 mm increase in the measure is thought to be linked to a 4% increase in the risk of EPE [19]. The optimal threshold for detection is associated with the balance between the sensitivity and the specificity and takes values from 6 mm to 20 mm [18–22]. In the current study, LCC measurements performed independently by two radiologists reveal moderate performances and very similar optimal cut-off values (i.e. 14.5 mm and 15.8 mm) that lead to moderate sensitivity and specificity in the detection of EPE positive tumors. LCC estimates from MR images offer good inter-observer agreements. The results of the current study is in consistent with the previous studies that reported fair performance with good interobserver agreement rates [18–22]. We recomended the LCC cut off value of 14.5 mm for detecting EPE in prostate cancer.

For grading EPE for an EPE positive prostate tumor, the radial distance of EPE, described as the length of tumor protrusion perpendicular to the outer margin of the prostatic stroma, has been voted as a beneficial metric. However, several studies demonstrate that if used as a continuous metric, the radial distance determined from the radical prostatectomy specimens is insignificantly correlated with prognosis. Besides, an increase in the metric is shown to be signiﬁcantly associated with an increase in the risk of biochemical recurrence [4,24,25]. Significant correlations can be obtained when the metric is converted into its categorical form by performing thresholding, and the median of the radial distance from a large population of prostate is recommended as an optimal threshold [24,26]. In agreement with these studies, an optimal threshold of 1.0 mm is determined in the current study and later used to categorize the high-grade and low-grade EPE positive prostate tumors. 

In the current study, LCC estimate from multi-parametric MR images provides fair diagnostic performance and reveals moderate sensitivity and specificity in discriminating high-grade from low-grade EPE positive tumors with the optimal LCC cut-offs of values of 20.0 mm and 18.5 mm. The performance of the MR-estimated LCC in discriminating the EPE-positive patients according to the amount of EPE has not been previously studied. Nevertheless, the distinct cut-off values for diagnosing any EPE (whether focal-low-grade or established-high grade EPE) and established-high grade EPE has been published only in one previous study that reports the optimal thresholds of MR estimated LCC as 6 mm for detecting EPE and 10 mm for diagnosing established-high grade EPE [20]. When compared to that work, higher optimal LCC thresholds are reported for the detection and the diagnosis in the current work. This difference can be explained by two different perspectives. Firstly, LCC is obtained for an index lesion localized within the entire prostate gland in the current study, while it is measured for the dominant lesion localized within each of the lobes of the prostate gland in [20]. Secondly, while the cut-off value of pathological RD was taken as 0.5 mm for classifying patients as focal versus established in [20]., the current study reports the cut-off value of 1.0 mm for pathological RD to discriminate the high-grade EPE from the low-grade EPE. The median value of the pathological RD obtained from a patient population is recommended for use as a cut-off for discriminating the high-grade from the low-grade EPE positive tumors. A threshold value of 1.0 mm is obtained in the current study that is in accordance with some previous studies one of which enrolls the largest study cohort of EPE positive tumors and utilizes 1.0 mm cut-off as the optimal threshold for RD that is signiﬁcantly associated with the increase in BCR risk [24–26]. We recommend the LCC cut off value of 18.5 mm for distinguishing low grade EPE from high grade EPE.

The results of the current study note a moderate correlation between pathological RD and the MR estimated LCC, while a poor correlation is demonstrated between pathological RD of EPE and pathological LCC. This is an unexpected finding that required an explanation on whether there is a significant difference between pathological LCC and MR determined LCC measurements according to the EPE status. We observed that pathological LCC and MR determined LCC measurements are highly correlated in all groups when patients are classified according to EPE status. However, this correlation is much stronger in patients with a high amount of EPE than in patients with a low amount of EPE (Table 3 ). Bakır et al. reports that, in the low ISUP grading group, the pathological LCC and MR estimated LCC measurements are less concordant, and statistical results involving the LCC and EPE relationship for pathological LLC and MR estimated LCC measurements are diverging [23]. Considering more aggressive tumors with higher ISUP grading group shows a higher tendency to extension beyond the prostatic capsule; the findings of the current study is in accordance with the literature. LCC measurements from MR images may be overestimated or underestimated for less aggressive prostate tumors, and this might cause lower correlations with LCC estimates from radical prostatectomy specimens. Tumor aggressiveness may play a role in establishing the relationship between LCC and EPE. 

There are some limitations of the current study. The study has a retrospective design, and this may lead to some selection bias for the prostate tumors taken into analysis. The study dataset covers a large number of prostate tumors, but the number of EPE positive tumors in the dataset is limited. Consequently, the results reported may not be generalized well for the EPE positive tumors. Although MR images of the prostate tumors are with high quality, the length of capsular contact may be under- or over-estimated especially for less aggressive tumors due to resolution margins of the recent MR imaging technology. 

In conclusion, multi-parametric MR images deliver reliable estimates of the length of capsular contact for prostate tumors that can be used in detecting and grading extraprostatic extension for the tumors in local staging of cancer and selection of appropriate surgical plan. We suggested further prospective studies with larger study cohorts to clarify potential benefits, and computational tools are needed to be developed to promote the use of MR-derived length of capsular contact in clinical practice. 

## Funding

This research received no specific grant from any funding agency in the public, commercial, or not-for-profit sectors. 

## Informed consent

Institutional review board approval and informed consent are secured for this retrospective study (The relevant review board and approval code is 2020.283.IRB2.074).
